# Serum Metrnl is associated with the presence and severity of coronary artery disease

**DOI:** 10.1111/jcmm.13915

**Published:** 2018-11-05

**Authors:** Zheng‐Xia Liu, Hui‐Hong Ji, Min‐Peng Yao, Li Wang, Yue Wang, Ping Zhou, Ying Liu, Xi‐Feng Zheng, Hui‐Wei He, Lian‐Sheng Wang, Wei Gao, Xiang Lu

**Affiliations:** ^1^ Department of Geriatrics The Affiliated Sir Run Run Hospital of Nanjing Medical University Nanjing China; ^2^ Key Laboratory for Aging & Disease Nanjing Medical University Nanjing China; ^3^ Laboratory of Geriatrics The Second Affiliated Hospital of Nanjing Medical University Nanjing China; ^4^ Department of Internal Medicine The Hospital of HoHai University Nanjing China; ^5^ Department of Cardiology The First Affiliated Hospital of Nanjing Medical University Nanjing China

**Keywords:** coronary artery disease, Metrnl, presence, severity

## Abstract

Meteorin‐like (Metrnl) is a novel adipokine that is highly expressed in white adipose tissue. Metrnl stimulates energy expenditure and improves glucose tolerance in rodents. However, whether Metrnl plays a role in coronary artery disease (CAD) remains to be elucidated. The present study aimed to investigate the association of serum Metrnl with CAD in Chinese patients. A total of 193 patients with CAD and 156 control subjects were enrolled in this study. Serum Metrnl concentration was measured by enzyme‐linked immunosorbent assay. Anthropometric phenotypes, fasting glucose, serum lipids, and inflammatory cytokines were measured. Serum Metrnl was lower in CAD patients when compared to those controls (132.41 vs 173.17 pg/mL, *P* < 0.001). Serum Metrnl was negatively correlated with metabolic parameters, including body mass index, total cholesterol, and low‐density lipoprotein cholesterol as well as inflammatory markers including high‐sensitivity C‐reactive protein, IL‐1β, and IL‐11 even after adjustment for potential confounding variables (*P* < 0.05). In multivariable logistic regression analyses, compared to those in the highest tertile of serum Metrnl levels, subjects in the lowest tertile had the highest risks for CAD (adjusted OR = 2.63, 95% CI = 1.46‐4.27, *P* = 0.001). After adjustment for potential confounding variables, serum Metrnl was also decreased as the number of stenosed vessels increased (*P* < 0.001). Furthermore, decreased Metrnl level was negatively correlated with the severity of CAD quantified by the Gensini score. This first case‐control study shows significant associations of serum Metrnl with the presence and severity of CAD, suggesting Metrnl might be a new promising therapeutic target for CAD.

## 1 INTRODUCTION

Coronary artery disease (CAD) remains the leading cause of morbidity and mortality in the adult population worldwide.^1^ Adipose tissue has been recognized as the largest endocrine and paracrine organ that produces hundreds of proteins contributing as enzymes, hormones, or growth factors in various pathophysiologic processes.^2^ These proteins are known under the term “adipokines,” for example, adiponectin, leptin, resistant, visfatin, retinol‐binding protein 4, etc., which are involved in the pathophysiology of obesity and insulin resistance and therefore have indirect effects on atherosclerosis.^3^, ^4^ For instance, adiponectin is an adipose tissue‐derived adipokine which has protective role in the initiation and progression of atherosclerosis through its anti‐inflammatory and antiatherogenic effects.^5^ Leptin exerts various atherogenic effects such as induction of endothelial dysfunction, stimulation of inflammatory reaction and oxidative stress, decrease in paraoxonase activity, platelet aggregation, etc.^6^ Leptin‐deficient and leptin receptor‐deficient mice are protected from arterial thrombosis and neointimal hyperplasia in response to arterial wall injury.^7^ Our previous studies indicated that visfatin, a pro‐inflammatory adipokine expressed markedly in visceral fat, may promote atherosclerosis through stimulating vascular inflammation.^8^, ^9^

Recently, Meteorin‐like (Metrnl), also known as Cometin and Subfatin, has been identified as a novel adipokine that is highly expressed in the subcutaneous white adipose tissue.^10^ Rao et al^11^ found that Metrnl was induced in white adipose tissue during acute cold exposure and in muscle after acute bouts of exercise or overexpression of Peroxisome proliferator‐activated receptor gamma coactivator 1‐alpha (PGC‐1α). Metrnl could promote beige/brown fat thermogenesis and improve glucose tolerance via activating IL‐4/IL‐13 signalling.^11^ Another study showed that Metrnl was downregulated in white adipose tissue after caloric restriction but was dramatically upregulated during white adipocyte differentiation and in the white adipose tissue of diet‐induced obese mice.^12^ Li et al^13^ demonstrated that adipocyte‐specific deficiency of Metrnl exacerbated insulin resistance induced by high‐fat diet, while adipocyte‐specific overexpression of Metrnl prevented insulin resistance induced by high‐fat diet or leptin deletion. Further experiments identified that Metrnl antagonized obesity‐induced insulin resistance by improving adipose function, including adipocyte differentiation, metabolism activation, and inhibiting adipose inflammation through the PPARγ pathway.^13^ A clinical study also showed that serum Metrnl level was decreased and negatively correlated with glucose level and insulin resistance in patients with diabetes.^14^ Chung et al^15^ found that circulating Metrnl level was inversely related to various cardiometabolic risk factors including body composition, components of metabolic syndrome. It has been well known that insulin resistance and hyperglycaemia are independent risk factors for CAD.^16^ Moreover, considering the central position of inflammation in the pathogenesis of atherosclerosis and Metrnl has an inhibitory role in inflammation, we hypothesized that decreased serum Metrnl levels might be correlated with CAD. Therefore, the present case‐control study aimed to investigate the association of serum Metrnl levels with the presence and severity of CAD in Chinese patients.

## 2 MATERIALS AND METHODS

### 2.1 Study population

The study population was composed of CAD patients and control subjects. The CAD patients were recruited from inpatients admitted to the Affiliated Sir Run Run Hospital and Second Affiliated Hospital of Nanjing Medical University because of angina pectoris or other symptoms or signs of cardiovascular disease. Acute myocardial infarction (AMI) was diagnosed if a patient had a cardiac troponin I level exceeding the 99th percentile of a normal reference population with ≥1 of the following: chest pain lasting >20 minutes, diagnostic serial electrocardiographic changes consisting of new pathologic Q waves, or ST‐segment and T‐wave changes.^17^ The control subjects were selected during the same period in the same hospital from the health examination centre. Considering it was unethical to perform coronary angiography to rule out the presence of asymptomatic CAD, the following inclusion criteria were used: no history of angina, no symptom or sign of other atherosclerotic vascular diseases. All subjects included in this study had no history of significant concomitant diseases, including severe hepatic or renal diseases, bleeding disorders, previous thoracic irradiation therapy, and malignant diseases. Hypertension was defined as resting systolic blood pressure (SBP) above 140 mmHg and/or diastolic blood pressure (DBP) above 90 mmHg or in the presence of active treatment with antihypertensive agents. Diabetes mellitus was defined as fasting blood glucose (FBG) >7.0 mmol/L or a diagnosis with diet adjustment or antidiabetic drug therapy. Dyslipidaemia was defined according to the guideline of the National Cholesterol Education Program (Adult Treatment Panel III). Written informed consent was obtained from each participant and this study was approved by the Ethics Committee of the Affiliated Sir Run Run Hospital and Second Affiliated Hospital of Nanjing Medical University.

### 2.2 Coronary angiography

Two cardiologists who were unaware of the patients included in this study assessed the angiograms. CAD was defined as luminal diameter narrowing estimated visually at least 50% in any epicardial coronary artery, including the left main coronary artery, left anterior descending, left circumflex, or right coronary artery. CAD patients were divided into single‐, double‐, and triple‐vessel disease subgroups according to the number of significantly stenosed vessels. The severity of CAD was assessed with the Gensini score system based on the degree of luminal narrowing and its geographic importance.^18^

### 2.3 Laboratory measurements

Venous blood sample was collected from each subject. The whole blood was separated into serum and cellular fractions within 2 hours by centrifugation at 3000 *g* for 10 minutes. The supernatant (serum) was collected and further centrifuged at 10 000 *g* for 15 minutes to completely remove the cell debris. The obtained serum was stored at −80°C before further analysis. Total cholesterol (TC), triglyceride (TG), high‐density lipoprotein cholesterol (HDL‐C), and low‐density lipoprotein cholesterol (LDL‐C) levels were measured enzymatically on a chemistry analyzer (Olympus AU5400; Chemical Ltd., Tokoy, Japan). Glucose levels were measured by a glucose oxidase method (Reagent kit; Diagnostic Chemicals Ltd., London, UK). High‐sensitivity C‐reactive protein (hs‐CRP) was determined using an enzyme‐linked immunosorbent assay kit (R&D systems, Minneapolis, MN, USA).

### 2.4 Inflammatory cytokine measurements

Inflammatory cytokines including IL‐1β, IL‐8, and IL‐11 were measured using the CBA Human Soluble Protein Detection Kit (BD Biosciences, San Jose, CA, USA) as previously described.^19^ Briefly, blood samples and standards were incubated with capture beads for 1 hour at room temperature in the dark. The phycoerythrin detection reagent was then added and incubated for 2 hours at room temperature in the dark. The samples were washed and the bead pellets were resuspended in washing buffer. The resuspended samples were then run on a flow cytometer (FACS Canto II; BD Biosciences). Two thousand events in the gated bead population were collected, and 5‐parameter data were saved for subsequent analysis using BD FCAP Array software. Serum concentrations were derived using the standard curve and expressed in pg/mL.

### 2.5 Serum Metrnl measurement

Serum Metrnl levels were measured by using an enzyme‐linked immunosorbent assay kit (R&D systems) according to the manufacturer's protocol. The intra‐ and interassay coefficients of variance were 2.59% and 3.55% respectively. The analytic sensitivity of the assays was 15.625 ng/mL.

### 2.6 Statistical analysis

Statistical analyses were performed using PASW 18.0 (IBM SPSS Inc., Chicago, IL, USA). Normality of distribution was assessed using the Kolmogorov‐Smirnov test. Data for age and LDL‐C were normally distributed parameters and presented as the mean ± SD, and comparisons were analysed by Student's *t* test. Skewed data, including SBP, DBP, TC, TG, HDL‐C, FBG, Cr, hs‐CRP, IL‐1β, IL‐8, IL‐11, Gensini score, and Metrnl, were expressed as median and quartile ranges, and comparisons were analysed by the Mann‐Whitney *U* test. Pearson chi‐squared test was used to compare qualitative variables represented as frequencies. The correlations between serum level of Metrnl and other variables were calculated using Spearman correlation coefficient and partial correlation coefficient adjusted for age, sex, smoking, alcohol intake, BMI, Cr, FBG, TC, TG, and LDL‐C, as appropriate. Univariate analysis and multivariate logistic regression analysis were taken to determine the variables that independently contributed to the presence of CAD. Odds ratios (ORs) and 95% confidence intervals (CIs) were calculated. Linear regression analysis was used to test for trend of the changes of serum Metrnl concentrations across the severity of coronary angiography (normal to triple‐vessel disease). Receiver operating characteristic (ROC) curve analysis was used to determine the optimum cut‐off level of Metrnl best predicting CAD. To assess the performance of predictive model of Metrnl for CAD, we calculated the concordance (*c*) statistics for discriminative ability and goodness of fit statistics for calibration. All tests were two‐sided and *P* < 0.05 was considered statistically significant.

To further explore the synergistic effect of Metrnl and classical risk factors on the risk of CAD, a 4 × 2 table approach was conducted to calculate OR, respective 95% CI, and two‐tailed *P*‐values. It was assumed that subjects unexposed to the traditional risk factors and with a Metrnl concentration above the cut‐off value have a certain background risk for CAD (defined as OR00, with the value assumed to be 1). OR01 refers to the relative risk for CAD among those unexposed to a risk factor with a Metrnl concentration below the cut‐off value when compared with those with neither a Metrnl concentration below the cut‐off nor exposure to risk factors. OR10 refers to the relative risk for CAD among those with a Metrnl concentration above the cut‐off value but exposure to an environmental risk factor when compared with those with neither a Metrnl concentration below the cut‐off value nor exposure to risk factors. OR11 is the ratio of the CAD risk among subjects exposed to risk factors with a Metrnl concentration below the cut‐off value when compared with the CAD risk among unexposed subjects with a Metrnl concentration above the cut‐off value. These ORs were used in the calculation of the synergy indexes (SIs), such that SI = (OR11‐1)/(OR10 + OR01‐2) and SIM = OR11/(OR10 × OR01); the relative excess risk due to interaction, RERI = OR11‐OR10‐OR01 + 1; and the attributable proportion of the disease due to interaction, AP = RERI/OR11.

## 3 RESULTS

### 3.1 Characteristics of the study participants

Table 1 presents the characteristics of the study population. The 349 participants (mean age 65.1 ± 9.2 years) included 193 patients with CAD and 156 control subjects. There were no statistically significant differences between CAD patients and control subjects with respect to age, proportion of male, body mass index (BMI), rates of smoking and alcohol intake, rates of hypertension and hyperlipidaemia, TC, HDL‐C, and creatine (Cr) levels. However, CAD patients, compared with control subjects, had higher rates of diabetes, higher levels of SBP and DBP (*P* < 0.01); higher levels of TG and LDL‐C (*P* < 0.03); higher levels of FBG, hs‐CRP, and IL‐1β (*P* < 0.01); higher rates of taking antihypertension drugs, antihyperglycaemic drugs, and hypolipidaemic drugs (*P* < 0.001); and lower level of IL‐11 (*P* = 0.005). In addition, the serum levels of Metrnl were lower in CAD patients when compared to the control subjects (median: 132.41 pg/mL vs 173.17 pg/mL, *P* < 0.001).

**Table 1:** Characteristics of the participants

Variables	Control (n = 156)	CAD (n = 193)	*P*‐value
Age (years)	64.4 ± 8.1	65.7 ± 10.0	0.191
Male, n (%)	90 (57.7)	121 (62.7)	0.379
BMI (kg/m^2^)	24.3 (23.4‐25.4)	24.8 (23.4‐26.4)	0.178
Smoking, n (%)	70 (44.9)	93 (48.2)	0.590
Alcohol, n (%)	21 (13.5)	38 (19.7)	0.151
Hypertension, n (%)	106 (67.9)	142 (73.6)	0.286
Diabetes, n (%)	33 (21.2)	69 (35.8)	0.003
Hyperlipidaemia, n (%)	74 (47.4)	103 (53.4)	0.283
Antihypertensive therapy, n (%)	94 (60.3)	137 (80.0)	<0.001
Antihyperglycaemic therapy, n (%)	25 (16.0)	63 (32.6)	<0.001
Antihyperlipidaemic therapy, n (%)	51 (32.7)	99 (51.3)	<0.001
SBP, mmHg	133.5 (125.0‐145.0)	140.0 (130.0‐154.5)	0.001
DBP, mmHg	75.5 (70.0‐86.0)	85.0 (72.0‐93.5)	0.003
TC (mmol/L)	4.39 (3.75‐5.12)	4.59 (3.85‐5.32)	0.117
TG (mmol/L)	1.23 (0.96‐1.55)	1.38 (1.01‐2.01)	0.013
LDL‐C (mmol/L)	2.54 ± 0.77	2.74 ± 0.89	0.027
HDL‐C (mmol/L)	1.09 (0.98‐1.33)	1.07 (0.91‐1.29)	0.171
FBG (mmol/L)	5.97 (5.33‐7.02)	6.32 (5.43‐8.02)	0.004
Cr (μmol/L)	77.26 (72.53‐83.99)	77.64 (72.53‐85.66)	0.378
hs‐CRP (mg/L)	1.05 (0.80‐3.00)	5.00 (1.30‐12.00)	<0.001
Metrnl (pg/mL)	173.17 (142.27‐200.40)	132.41 (107.44‐167.45)	<0.001
IL‐1β^a^ (pg/mL)	0.99 (0.50‐1.83)	1.41 (0.89‐2.91)	0.002
IL‐8^a^ (pg/mL)	11.07 (6.24‐20.78)	11.58 (5.80‐21.71)	0.786
IL‐11^a^ (pg/mL)	125.57 (74.44‐227.28)	74.56 (27.20‐225.20)	0.005

Data are mean ± SD, median with interquartile range in parenthesis, or number with percentage in parenthesis.

CAD: coronary artery disease; BMI: body mass index; SBP: systolic blood pressure; DBP: diastolic blood pressure; TC: total cholesterol; TG: triglyceride; HDL‐C: high‐density lipoprotein cholesterol; LDL‐C: low‐density lipoprotein cholesterol; FBG: fasting blood glucose; Cr: creatine; hs‐CRP: high‐sensitivity C‐reactive protein.

a The concentrations of IL‐1β, IL‐8, and IL‐11 were measured in 48 patients with CAD and 54 control subjects.

### 3.2 Association of serum Metrnl with clinical parameters

We next investigated the relationship of serum Metrnl levels with various clinical parameters (Table S1). Serum Metrnl levels were negatively associated with BMI (*r* = −0.274; *P* = 0.001), TC (*r* = −0.309; *P* < 0.001), and LDL‐C (*r* = −0.224; *P* = 0.005) in the CAD group. The correlations remained statistically significant (*P* < 0.05) after adjustments for age, sex, smoking, alcohol intake, BMI, Cr, FBG, TC, TG, and LDL‐C, as appropriate. Serum Metrnl levels were also negatively associated with BMI (*r* = −0.171; *P* = 0.018), Cr (*r* = −0.156; *P* = 0.031), and LDL‐C (*r* = −0.147; *P* = 0.041) in the control group; however, the correlations lost significance after adjustment for the aforementioned variables. We further investigated the possible interferences of medications with Metrnl levels. Although there was slight increase in serum Metrnl concentrations in patients received antihyperglycaemic therapy, the difference did not reach statistical significance (median: 151.48 pg/mL vs 142.69 pg/mL, *P* = 0.119). Similar results were observed in patients received antihypertensive therapy (*P* = 0.126) and antihyperlipidaemic therapy (*P* = 0.299).

**Figure 1:**
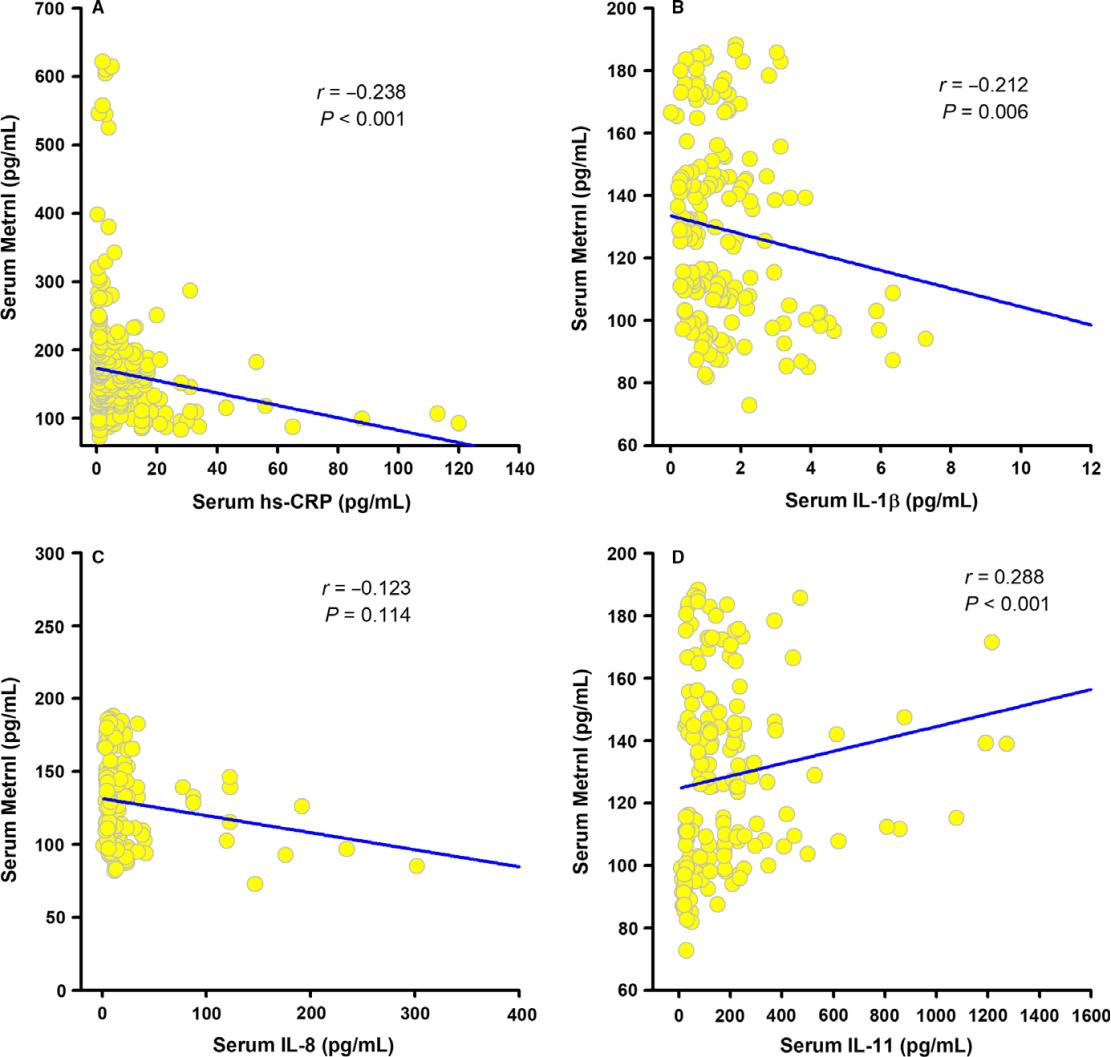
Correlation of serum Metrnl with inflammatory markers. Spearman correlation coefficient was used to analyse the association of serum Metrnl levels with hs‐CRP (A), IL‐1β (B), IL‐8 (C), and IL‐11 (D) in patients with CAD (n = 48) and control subjects (n = 54)

We further analysed the relationship between serum Metrnl and inflammatory markers. As shown in Figure 1, serum Metrnl levels were negatively correlated with hs‐CRP (*r* = −0.238; *P* < 0.001) and IL‐1β (*r* = −0.212; *P* = 0.006), but positively correlated with anti‐inflammatory cytokine IL‐11 (*r* = 0.288; *P* < 0.001). The association of Metrnl with hs‐CRP (*r* = −0.141; *P* = 0.009), IL‐1β (*r* = −0.240; *P* = 0.002), and IL‐11 (*r* = 0.178; *P* = 0.025) remained statistically significant even after adjustments for age, sex, smoking, alcohol intake, diabetes, BMI, Cr, FBG, TC, TG, and LDL‐C.

### 3.3 Association of serum Metrnl level with the presence of CAD

**Figure 2:**
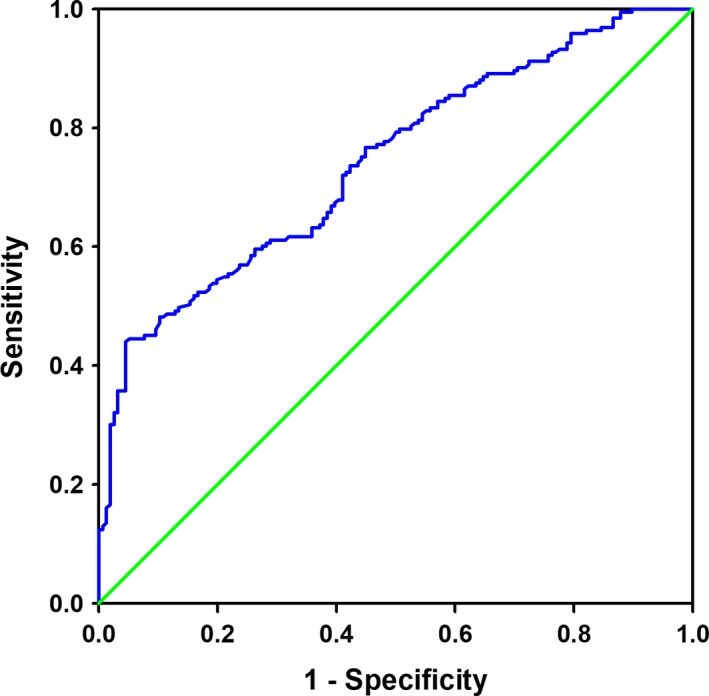
Receiver operating characteristic curves for the diagnostic accuracy of Metrnl for CAD

**Figure 3:**
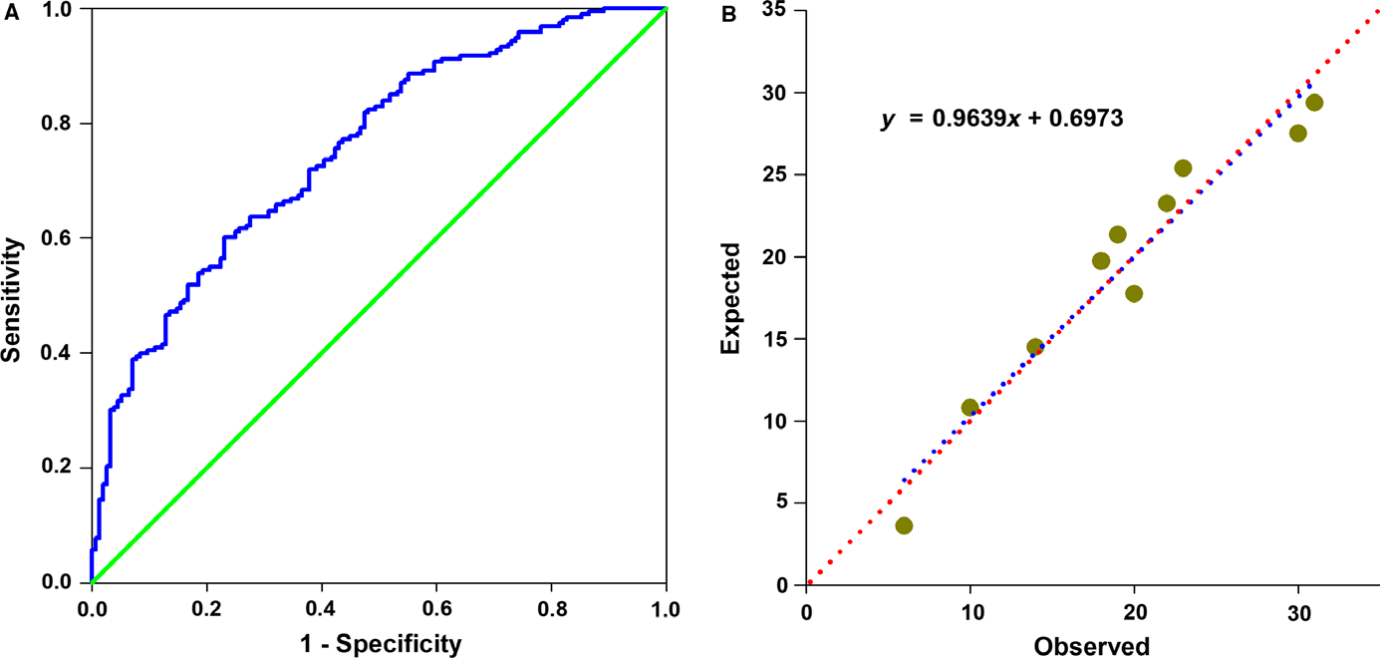
The discrimination and calibration of the predictive model of Metrnl for CAD. (A) Receiver operating characteristic (ROC) curve for the predicted probabilities without (red dot line) and with the marker Metrnl (blue dot line). (B) Scatter plot of predicted probabilities without and with the marker Metrnl

Univariate and multivariate logistic regression analyses showed that decreased serum Metrnl levels were significantly associated with the presence of CAD both before and after adjustment for potential confounders including age, sex, BMI, smoking, alcohol intake, hypertension, diabetes, hypeylipidaemia, TC, TG, LDL‐C, HDL‐C, FBG, and Cr (Table 2 and Table S2). Similar results were obtained by logistic regression using log‐transformed serum Metrnl as a continuous variable (adjusted OR = 0.85, 95% CI = 0.80‐0.90, *P* < 0.01). Finally, we conducted ROC curve analysis to identify the diagnostic accuracy of Metrnl for CAD. The optimal cut‐off value of Metrnl that predicted CAD was 123.5 pg/mL, with a sensitivity of 44.04% and a specificity of 95.51% (area under the curve = 0.742, 95% CI = 0.69‐0.79, *P* < 0.0001) (Figure 2). We further analysed the discrimination and calibration of our predictive model. As show in Figure 3A, the *c* statistics (equal to area under the curve) is 0.753, indicating good discriminative ability. The calibration curves (Figure 3B) and the results of Hosmer‐Lemeshow test (χ^2^ = 6.26, *P* = 0.618) also indicate reasonable fit of our model.

**Table 2:** Associations of serum Metrnl with the presence of CAD

	Tertile 2 vs Tertile 3		Tertile 1 vs Tertile 3	
	OR (95% CI)	*P*‐value	OR (95% CI)	*P*‐value
Crude model	2.49 (1.87‐3.32)	<0.001	2.56 (1.50‐4.35)	0.001
Adjusted model	2.54 (1.86‐3.47)	<0.001	2.63 (1.46‐4.72)	0.001

The adjusted model included age, gender, BMI, smoking, alcohol intake, hypertension, diabetes, hypeylipidaemia, TC, TG, LDL‐C, HDL‐C, FBG, and Cr.

OR: odds ratio; CI: confidence interval.

### 3.4 Association of serum Metrnl level with the severity of CAD

**Figure 4:**
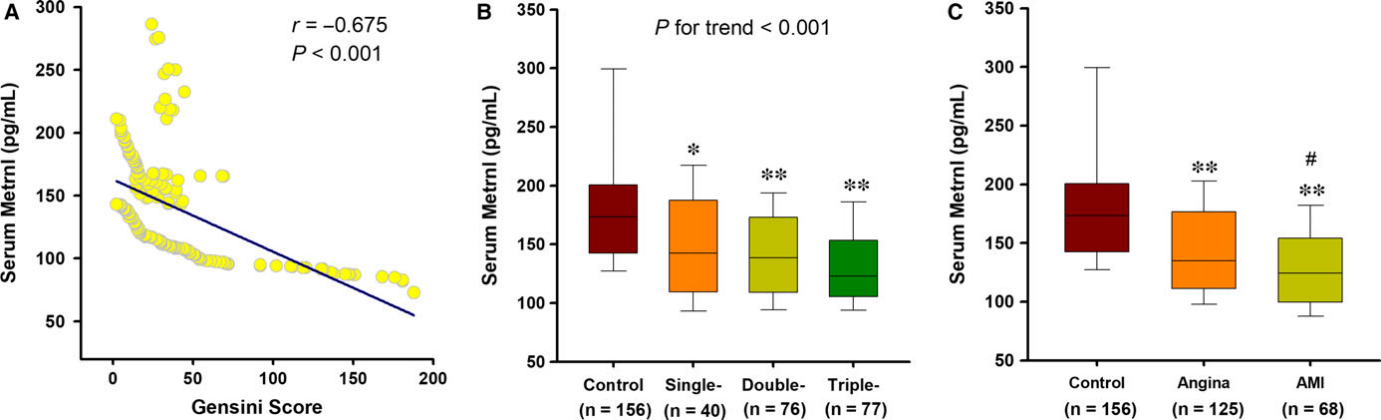
Association of Metrnl with the severity of CAD. (A) Spearman correlation coefficient was used to analyse the correlation between serum Metrnl levels and Gensini scores. (B) Serum Metrnl level decreases as the number of affected vessels increases. The analysis was adjusted for age, sex, BMI, smoking, alcohol intake, hypertension, diabetes, hypeylipidaemia, TC, TG, LDL‐C, HDL‐C, FBG, and Cr. The *P*‐value for test for trend of the changes of serum Metrnl concentrations across the severity of coronary angiography is given. *P < 0.01 vs Control; **P < 0.001 vs Control. (C) Changes of serum Metrnl in patients with acute myocardial infarction (AMI). **P < 0.001 vs Control; #*P* < 0.05 vs Angina

We further divided patients with CAD into three subgroups according to the number of stenosed vessels. As the number of affected vessels increased, the Gensini score significantly increased (median: 20.0 vs 22.5 vs 32.5, *P* = 0.035). Spearman correlation analysis demonstrated negative correlations of Metrnl with the severity of CAD, quantified by the Gensini score (*r* = −0.675; *P* < 0.001) (Figure 4A). Linear regression analysis indicated that serum Metrnl levels were decreased as the number of stenosed vessels increased (β = −0.267, *P* for trend <0.001) (Figure 4B). After adjustment for the aforementioned covariates, the trends of serum Metrnl levels across the severity of CAD remained statistically significant (β = −0.240, *P* for trend <0.001). We further investigated the changes of serum Metrnl in patients with AMI. Interestingly, serum Metrnl levels were also decreased in patients with AMI (Figure 4C) and negatively correlated with hs‐CRP (*r* = −0.403; *P* = 0.001).

### 3.5 Stratification analyses for the association of Serum Metrnl with the presence and severity of CAD

Previous studies revealed that Metrnl could antagonize insulin resistance and improve glucose tolerance in mice.^13^ We thus separated the subjects into 102 patients with diabetes and 247 patients without diabetes. However, we did not find significant difference of serum Metrnl between patients with diabetes and those without diabetes (median: 142.69 pg/mL vs 152.04 pg/mL, *P* = 0.081). After exclusion of diabetic patients, subjects in the lowest tertile compared with the highest tertile of serum Metrnl were associated with higher risks for CAD even after adjustment for the potential confounders (adjusted OR = 3.25, 95% CI = 1.61‐5.56, *P* < 0.001, Table 3). Similarly, the downward trend of serum Metrnl levels across the severity of CAD remained significant in patients without diabetes after adjustment for potential confounders (β = −0.231, *P* < 0.001, Figure S1). Moreover, we did not find significant difference of serum Metrnl between men and women in our study (median: 147.10 pg/mL vs 150.27 pg/mL, *P* = 0.366). Separating subjects into males and females also did not materially change the association of Metrnl with the presence and severity of CAD (Table 3 and Figure S2).

**Table 3:** Stratification analyses for the association of serum Metrnl with the presence of CAD

	Tertile 2 vs Tertile 3				Tertile 1 vs Tertile 3			
	Crude OR (95% CI)	*P*‐value	Adjusted OR (95% CI)	*P*‐value	Crude OR (95% CI)	*P*‐value	Adjusted OR (95% CI)	*P*‐value
Diabetes								
With diabetes	1.39 (0.49‐3.90)	0.538	1.75^a^ (0.42‐7.23)	0.440	1.91 (1.11‐3.29)	0.020	2.11^a^ (1.01‐4.45)	0.048
Without diabetes	2.65 (1.88‐3.74)	<0.001	2.68^a^ (1.87‐3.83)	0.001	3.08 (1.64‐5.79)	<0.001	3.25^a^ (1.61‐6.56)	<0.001
Gender								
Male	2.64 (1.89‐3.98)	<0.001	2.87^b^ (1.91‐4.31)	0.001	3.70 (1.82‐7.52)	<0.001	3.46^b^ (1.64‐7.33)	<0.001
Female	1.53 (0.68‐3.46)	0.303	1.76^b^ (0.57‐5.50)	0.328	2.17 (1.38‐3.40)	0.001	2.73^b^ (1.56‐4.77)	<0.001

OR: odds ratio; CI: confidence interval.

a The adjusted model included age, gender, BMI, smoking, alcohol intake, hypertension, hypeylipidaemia, TC, TG, LDL‐C, HDL‐C, FBG, and Cr.

b The adjusted model included age, BMI, smoking, alcohol intake, hypertension, diabetes, hypeylipidaemia, TC, TG, LDL‐C, HDL‐C, FBG, and Cr.

### 3.6 Interaction between Metrnl and classical risk factors

As shown in Tables 4 and 5, regarding the conventional risk of subjects unexposed to both classical risk factor and Metrnl risk (reference category) as being 1.0, the ORs estimating the effect of joint exposure to Metrnl and sex (male was defined as risk gender), BMI, TC, LDL‐C, and Cr were significantly higher than the ORs estimating the effect of each factor in the absence of the other. A positive association between Metrnl concentration and sex was found (SI = 2.17, SIM = 1.58, and AP = 0.43), and in these groups, the proportion of CAD attributable to the interaction between Metrnl and sex was as high as 43%. Risk‐associated Metrnl concentration interacted with BMI in predicting CAD (SI = 2.25, SIM = 1.63, and AP = 0.44). The risk provided by TC was found to be positively reinforced by Metrnl (SI = 1.11, SIM = 0.77, AP = 0.08) in this study. Moreover, LDL‐C and Metrnl concentration interacted significantly in predicting the onset of CAD, and this combination accounted for 34% of the CAD cases (SI = 1.78, SIM = 1.31, and AP = 0.34).

**Table 4:** Synergistic effect of Metrnl and classical risk factors in CAD patients and controls

Classical risk	Metrnl	CAD	Controls	OR (95% CI)	*P*‐value
Sex					
0	0	42	64	1	–
0	1	66	85	1.18 (0.71‐1.96)	0.514
1	0	55	5	2.56 (1.84‐3.57)	<0.001
1	1	30	2	4.78 (2.28‐10.04)	<0.001
BMI					
0	0	43	65	1	–
0	1	65	84	1.17 (0.71‐1.94)	0.542
1	0	54	5	2.54 (1.82‐3.53)	<0.001
1	1	31	2	4.84 (2.31‐10.15)	<0.001
TC					
0	0	76	120	1	–
0	1	29	32	1.74 (0.98‐3.11)	0.060
1	0	19	1	3.11 (1.58‐6.12)	0.001
1	1	66	6	4.17 (2.68‐6.48)	<0.001
LDL‐C					
0	0	26	43	1	–
0	1	82	106	1.28 (0.73‐2.25)	0.393
1	0	60	5	2.71 (1.92‐3.82)	0.001
1	1	25	2	4.55 (2.13‐9.72)	<0.001
Cr					
0	0	5	15	1	–
0	1	103	134	2.31 (0.81‐6.55)	0.117
1	0	5	2	2.74 (1.05‐7.18)	0.040
1	1	80	5	3.63 (2.31‐5.71)	<0.001

CAD: coronary artery disease; OR: odds ratio; CI: confidence interval.

**Table 5:** The indexes of the synergistic effect between Metrnl and classical risk factors

	SI	SIM	RER1	AP
Sex‐Metrnl	2.17	1.58	2.04	0.43
BMI‐Metrnl	2.25	1.63	2.13	0.44
TC‐Metrnl	1.11	0.77	0.32	0.08
LDL‐C‐Metrnl	1.78	1.31	1.56	0.34
Cr	0.86	0.01	−1.31	−1.52

SI: Rothman's synergy index for an interaction; SIM: Khoury's synergy index for an interaction; RERI: relative excess risk due to an interaction; AP: proportion of disease attributable to an interaction.

## 4 DISCUSSION

The main finding of our study is that serum Metrnl, a novel adipokine with protective effect on insulin resistance, was strongly associated with CAD in a Chinese population. Among 349 subjects enrolled in our study, patients with the lowest tertile of serum Metrnl level were associated with nearly 1.5‐fold increase in the risk for CAD when compared to those with the highest tertile. Another interesting finding of the present study is that serum Metrnl level was also associated with the severity of CAD.

Metrnl is a novel‐secreted protein homologous to the neurotrophin Metrn.^12^, ^20^ As an adipokine, Metrnl has been shown to play important roles in energy expenditure and insulin resistance in rodents.^11^, ^13^ Rao et al^11^ showed that Metrnl expression was induced in adipose tissue upon acute cold exposure at 4°C and in muscle after an acute bout of concurrent exercise. Increased Metrnl promoted browning of white adipose tissue via an eosinophil‐dependent increase in IL4 expression and M2 macrophage activation, and thus stimulated energy expenditure and improved glucose tolerance.^11^ Li et al^13^ demonstrated that overexpression of Metrnl in adipocytes upregulated PPARγ expression, which in turn inhibited adipose inflammation, enhances adipocyte differentiation, activates lipid metabolism, and ultimately reduced insulin resistance. Recent clinical study also found that serum Metrnl level was significantly lower in patients with newly diagnosed diabetes mellitus and was negatively correlated with the serum glucose level and insulin resistance.^14^ These results suggest that decreased Metrnl might be a trigger of insulin resistance and subclinical inflammation, leading to the development of diabetes and cardiovascular diseases. Our present study supports these assumptions and shows that decreased serum Metrnl concentrations were not only associated with the risk for CAD but also correlated with the severity of CAD. To exclude the influence of diabetes mellitus on the relationship between Metrnl and CAD, we further investigated the association of Metrnl with CAD in nondiabetic subjects. Notably, the effect of Metrnl on CAD was larger after exclusion of diabetes mellitus (adjusted OR, 3.25 vs 2.63). It has been reported that Metrnl could improve insulin sensitivity and was correlated with metabolic parameters, including fasting glucose, fasting insulin, postload 2 hours glucose, HbA1c, and lipid levels.^21^ We thus hypothesized that the role of Metrnl as an insulin sensitizer might affect the predictive value of Metrnl for CAD in patients with diabetes. Although no significant correlation of Metrnl with fast glucose level was found in patients with (*r* = −0.08; *P* = 0.398) or without (*r* = −0.04; *P* = 0.971) diabetes, further clinical studies with larger sample on patients with diabetes are needed to confirm the predictive value of Metrnl for cardiovascular diseases.

Another interesting finding of our study is that serum Metrnl concentrations were independently inversely correlated with metabolic parameters, including BMI, TC, and LDL‐C. It has been long known that metabolic syndrome is associated with increased risk of CAD events.^21^ Both in vivo and in vitro studies have demonstrated that Metrnl could upregulate key transcription factors for adipocyte differentiation (PPARγ, C/EBPα) and lipid metabolism genes for lipid transport (FABP4, CD36), lipogenesis (ACC, FASN), lipolysis (Lipe, PNPLA), and lipid storage (Perilipin),^13^ indicating that Metrnl may mediate the link between metabolic disorder and CAD. Our results are consistent with those recently reported by Chung et al^15^ in a larger study regarding the association of Metrnl levels and metabolic syndrome. Another study also found that serum Metrnl levels were lower in patients with obesity compared to normal‐weight subjects, and significantly increased 12 months after laparoscopic sleeve gastrectomy, in correlation to improvements in glucose and lipid homoeostasis.^22^ However, our results go beyond those of Li et al's study,^13^ which showed no significant association of Metrnl with BMI, TC, glucose, or TG in human subjects undergoing a physical examination. Loffler et al^23^ reported that Metrnl expression in adipocytes was higher in obese children compared to lean children. The discrepancies in these studies might be due to several potential factors including study design, ethnicity, sample size, patient characteristics, and concomitant disease. However, considering the relatively modest *r* values in our study, further clinical studies with larger sample are needed to confirm these correlations.

The mechanism underlying the decreased circulating Metrnl in CAD remains unclear. To date, there has been no study that examined the effects of Metrnl in the development of atherosclerosis. Ushach et al^24^ showed that Metrnl was strongly induced in M2 macrophages and M2‐like macrophages. Metrnl could promote M2 macrophage activation associated with the suppression of inflammatory cytokines such as TNF‐α, IFN‐γ, and IL‐1β, and the increase in anti‐inflammatory genes such as IL‐10 and TGF‐β.^11^ It has been well known that pro‐inflammatory M1 macrophages participate in atherosclerosis initiation and progression while M2 macrophages are protective due to their anti‐inflammatory properties, presumably stabilizing the plaque.^25^ Considering that Metrnl is also highly expressed in heart and endothelial cells,^11^, ^24^ we speculate that decreased Metrnl might impair M2 polarization and potentiate inflammation of M1 macrophages, ultimately accelerating the development of atherosclerosis. Indeed, our study also found a negative correlation between serum Metrnl and pro‐inflammatory markers including hs‐CRP and IL‐1β as well as a positive correlation between Metrnl and anti‐inflammatory cytokine IL‐11. However, further studies using animal or cellular models are needed to delineate the effects and mechanisms of Metrnl in the pathological process of CAD and atherosclerosis.

Our study should be interpreted within the context of its limitations. The cross‐sectional design does not allow causal inference. Secondly, the relatively small sample size may underpower the results of our study. Thirdly, the CAD patients of our study were actually from a referral population. This might cause the possibility of selection bias and confound the results. In addition, as we only investigated Metrnl levels in Chinese patients, our findings need to be confirmed in other ethnicities.

## 5 CONCLUSIONS

In summary, our first case‐control study shows significant associations of serum Metrnl with the presence and severity of CAD. Although further prospective and interventional studies are necessary to investigate whether decreased Metrnl may participate in the development of atherosclerosis, our findings provide novel insights into the potential role of Metrnl in CAD.

## CONFLICT OF INTEREST

The authors declare that they have no competing interests.

## ETHICS APPROVAL AND CONSENT TO PARTICIPATE

Written informed consent was obtained from each participant and this study was approved by the Ethics Committee of the Affiliated Sir Run Run Hospital and Second Affiliated Hospital of Nanjing Medical University.

## AUTHOR CONTRIBUTION

Wei Gao and Xiang Lu designed the study, interpreted the data, and contributed to critically revising the manuscript. Zheng‐Xia Liu, Hui‐Hong Ji, and Min‐Peng Yao completed the project, analysed the data, and wrote the manuscript. Li Wang and Yue Wang contributed to data collection and Metrnl measurement. Xi‐Feng Zheng, Hui‐Wei He, and Lian‐Sheng Wang contributed to recruitment of patients and clinical diagnosis of disease. All authors have approved the final article.

## Supporting information

Data
