# Supplementary material for: Serum Metrnl is associated with the presence and severity of coronary artery disease
Source: J Cell Mol Med. 2018 Nov 5;23(1):271–80. doi: 10.1111/jcmm.13915 (PMC6307872; doi:10.1111/jcmm.13915)
Supplement: Supplementary file 1 — Data [file JCMM-23-271-s001.docx]

**Figure S1**

**
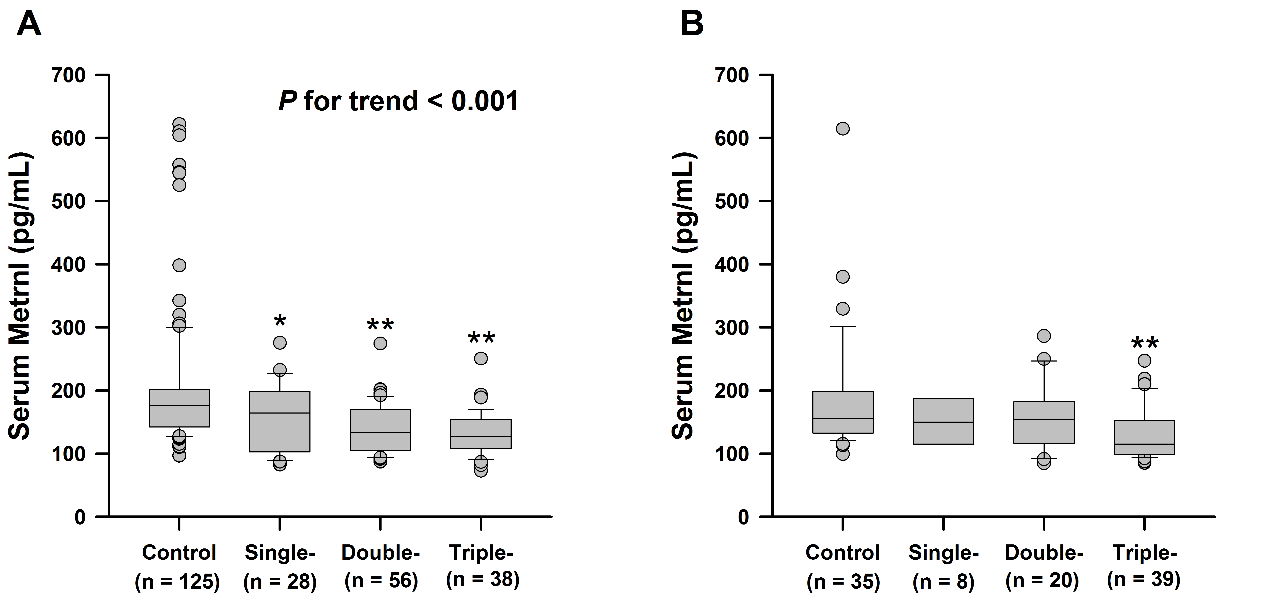
**

**Figure S1.** Association between serum Meteorin-like (Metrnl) and severity of coronary artery disease in patients without diabetes (A) and with diabetes (B). The *P* value for test for trend of the changes of serum Metrnl concentrations across the severity of coronary angiography is given. *, *P* < 0.01 *vs.* Control; **, *P* < 0.001 *vs.* Control.

**Figure S2**

**
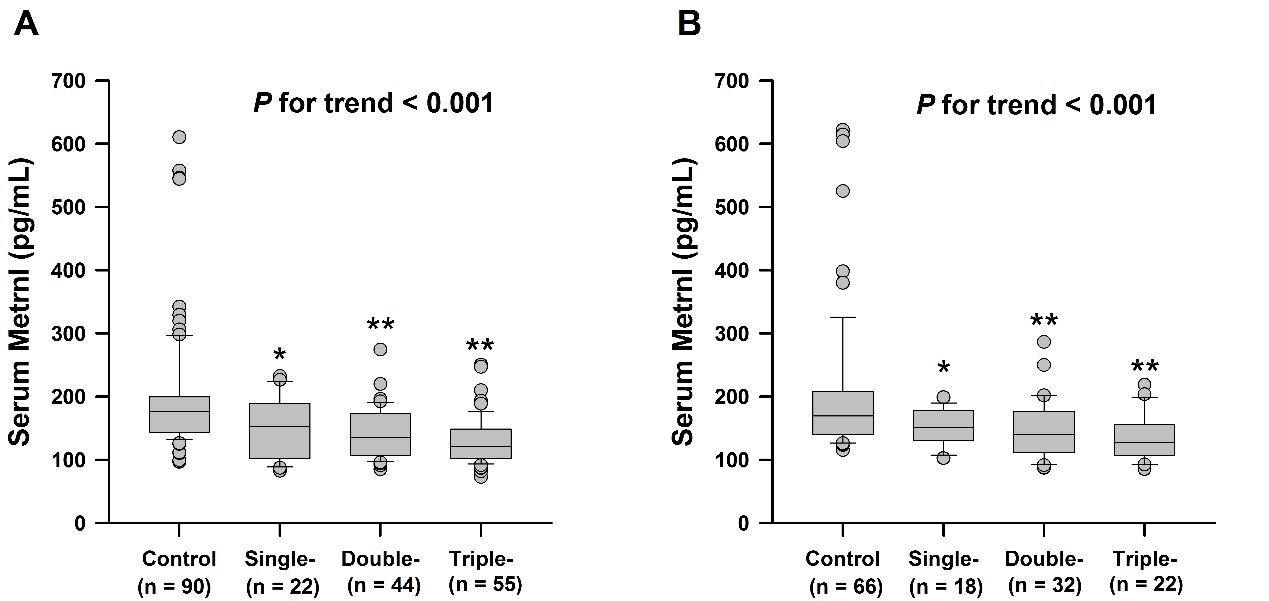
**

**Figure S2**. Association between serum Meteorin-like (Metrnl) and severity of coronary artery disease in men (A) and women (B). The *P* value for test for trend of the changes of serum Metrnl concentrations across the severity of coronary angiography is given. *, *P* < 0.01 *vs.* Control; **, *P* < 0.001 *vs.* Control.

**Table S1. Spearman’s correlation of serum Metrnl with clinical parameters**

| **Variables** | **CAD (n = 193)** | ***P* value** | **Control (n = 156)** | | ***P* value** |
| --- | --- | --- | --- | --- | --- |
| Age | - 0.073 | 0.365 | | 0.025 | 0.726 |
| BMI | - 0.274 | 0.001 | | - 0.171 | 0.018 |
| SBP | - 0.104 | 0.150 | | - 0.118 | 0.141 |
| DBP | - 0.136 | 0.053 | | - 0.116 | 0.148 |
| TC | - 0.309 | < 0.001 | | 0.147 | 0.065 |
| TG | - 0.126 | 0.116 | | - 0.083 | 0.254 |
| LDL-C | - 0.224 | 0.005 | | - 0.147 | 0.041 |
| HDL-C | - 0.044 | 0.585 | | 0.098 | 0.174 |
| FBG | - 0.070 | 0.387 | | - 0.097 | 0.181 |
| Cr | - 0.006 | 0.936 | | - 0.156 | 0.031 |

CAD, coronary artery disease; BMI, body mass index; SBP, systolic blood pressure; DBP, diastolic blood pressure; TC, total cholesterol; TG, triglyceride; HDL-C, high-density lipoprotein cholesterol; LDL-C, low-density lipoprotein cholesterol; FBG, fasting blood glucose; Cr, creatine.

**Table S2. Univariate analysis for the risk of CAD**

| **Variables** | **OR** | **95% CI** | ***P* value** |
| --- | --- | --- | --- |
| Age | 1.02 | 0.99 - 1.04 | 0.191 |
| Male | 1.23 | 0.80 - 1.90 | 0.342 |
| BMI | 1.06 | 0.96 - 1.16 | 0.278 |
| Smoking | 1.14 | 0.75 - 1.75 | 0.537 |
| Alcohol | 1.58 | 0.88 - 2.82 | 0.125 |
| Hypertension | 1.31 | 0.83 - 2.09 | 0.250 |
| Diabetes | 2.07 | 1.28 - 3.37 | 0.003 |
| Hyperlipidemia | 1.27 | 0.83 - 1.94 | 0.271 |
| TC | 1.23 | 1.01 - 1.50 | 0.045 |
| TG | 1.07 | 0.93 - 1.25 | 0.348 |
| LDL-C | 1.33 | 1.03 - 1.72 | 0.028 |
| HDL-C | 1.03 | 0.93 - 1.14 | 0.546 |
| FBG | 0.99 | 0.98 - 1.01 | 0.467 |
| Cr | 1.02 | 0.99 - 1.04 | 0.187 |
| hs-CRP | 1.18 | 1.12 - 1.25 | < 0.001 |
| Metrnl (continuous) | 0.98 | 0.97 - 0.99 | < 0.001 |
| Metrnl (ranked) | 0.40 | 0.30 - 0.53 | < 0.001 |

OR, odds ratio; CI, confidence interval; CAD, coronary artery disease; BMI, body mass index; TC, total cholesterol; TG, triglyceride; HDL-C, high-density lipoprotein cholesterol; LDL-C, low-density lipoprotein cholesterol; FBG, fasting blood glucose; Cr, creatine; hs-CRP, high‐sensitivity C‐reactive protein.
